# Thirty years of percutaneous epicardial access: reflections on the evolution of ventricular tachycardia substrate mapping and ablation

**DOI:** 10.1093/europace/euag263

**Published:** 2026-10-13

**Authors:** Mauricio Scanavacca

**Affiliations:** Arrhythmia Unit, Instituto do Coração (InCor), Hospital das Clínicas, Faculdade de Medicina, Universidade de São Paulo, São Paulo, Brazil

Scientific innovations often appear to emerge suddenly, becoming associated with a landmark publication or a novel technique. More often, they culminate from years of observation, unsuccessful attempts, and unanswered questions. The development of percutaneous epicardial access is one such example.

Its history did not begin in the electrophysiology laboratory. It began several years earlier in the operating room. The surgical programme for the treatment of sustained ventricular tachycardia (VT) at the Heart Institute (InCor), University of São Paulo Medical School, started in the late 1970s, based on the map-guided endocardial resection technique pioneered by Josephson, Harken, and Horowitz at the University of Pennsylvania.^1^

We soon encountered limitations familiar to other surgical groups, mainly in patients with Chagas disease. The clinical tachycardia was not always reproducibly induced; induced VTs were often rapid, apparently unrelated to the spontaneous arrhythmia, or degenerated into ventricular fibrillation. Complete circuit mapping and a reliable endpoint could therefore be difficult. This uncertainty led towards more empirical, extensive anatomical substrate modification, increasing the complexity and morbidity of surgery in an already diseased ventricle.

Recognizing these limitations, Professors Eduardo Sosa and Miguel Barbero-Marcial launched an ambitious project to introduce the intraoperative strategy developed by Mickleborough, Downar, and colleagues in Toronto, based on multi-electrode endocardial and epicardial mapping.^2^ Despite initial enthusiasm, technical difficulties prevented reliable implementation, and the project was discontinued.

Meanwhile, catheter ablation of VT was emerging as an alternative to surgery. Early experience, particularly from the French school led by Guy Fontaine, used endocardial activation and pace mapping to identify the presumed origin or exit, followed by DC shock ablation.^3^ Although effective in selected patients, the extensive lesions produced by DC shocks provided limited evidence that the target was a critical component of the re-entrant circuit.

The subsequent combination of entrainment mapping and radiofrequency (RF) ablation represented an important conceptual advance. Stevenson and colleagues demonstrated that pacing responses during post-infarction VT could identify sites functionally involved in the re-entrant circuit and distinguish them from adjacent bystanders, allowing relatively small RF lesions to interrupt re-entry at mechanistically defined targets. VT ablation had therefore become not only less invasive than surgery, but increasingly selective.^4^

Our initial experience using endocardial RF ablation in patients with chronic Chagas cardiomyopathy produced disappointing results despite apparently appropriate mapping. At the time, these failures raised a simple but important question: where were we failing? Was it because we did not understand the circuit, or because it was located in a region we could not reach?

In 1994, during a visit to Dr. Melvin Scheinman's laboratory at the University of California, San Francisco, I met Dr. Michael Lesh, who was working with one of the first multi-electrode basket catheters capable of recording simultaneously from multiple endocardial sites. Around the same period, I learned that Dr. Warren Jackman was investigating epicardial mapping with small multipolar catheters advanced through the coronary venous system. These developments suggested that simultaneous endocardial and epicardial mapping could soon be performed percutaneously and that VT investigation could move from the operating room to the electrophysiology laboratory. Upon my return to Brazil, discussions with Professor Eduardo Sosa and colleagues at InCor led us to explore ways to gain direct access to the pericardial space.^5,6^

In November 1995, at the annual scientific meeting of the Department of Arrhythmias and Electrophysiology of the Brazilian Society of Cardiology in Brasília, we presented our initial experience with endocardial RF ablation of sustained VT in chronic Chagas cardiomyopathy. Only approximately 25% of patients remained free from recurrence after one year. More important to us, however, were three patients in whom we had performed percutaneous epicardial mapping after unsuccessful endocardial procedures and before subsequent cardiac surgery. In one, the re-entrant circuit was clearly localized to the epicardial surface of the left ventricle. The observation suggested a possible explanation for some failures of endocardial ablation and, more importantly, a new way to investigate and treat VT without surgery.^5,6^

The presentation was met with silence and understandable scepticism. Yet, a memorable historical coincidence deserves mention: Josep Brugada was among those in the audience. Years later, epicardial mapping would become central to defining and treating the arrhythmogenic substrate of Brugada syndrome.^7^

Seen from today's perspective, the major innovation was not the recognition that ventricular arrhythmias could involve the epicardium; this was already known from surgical experience. The challenge was to regain access to the epicardial surface without surgery. The description of nonsurgical transthoracic pericardial access in 1996 brought epicardial exploration into the electrophysiology laboratory. What began as mapping soon evolved into an endocardial–epicardial strategy and direct epicardial RF ablation for recurrent VT.^8^

Chagas cardiomyopathy was the clinical catalyst, but the implications proved much broader. Even in ischaemic cardiomyopathy, in which most post-infarction VT circuits can be approached endocardially, epicardial mapping proved relevant in a smaller but meaningful subset. Its importance became clearer in arrhythmogenic and selected non-ischemic cardiomyopathies, in which substantial portions of the substrate may be subepicardial.^8^ Contemporary data from our group also illustrate how strongly epicardial access depends on the disease: it was used in 78% of Chagas procedures, vs. 15% in ischaemic and 31% in idiopathic dilated cardiomyopathy.^9,10^

Brugada syndrome represented a more profound conceptual advance. Epicardial mapping did not merely expose a target inaccessible from the endocardium; it revealed a distinct region of delayed, fractionated conduction over the right ventricular outflow tract and linked the surface ECG phenotype to a localized arrhythmogenic substrate.^7^ The expansion of this substrate after sodium-channel blockade and normalization of the Brugada pattern after ablation provided mechanistic evidence connecting abnormal conduction, repolarization, and ventricular fibrillation vulnerability. Thus, the epicardial approach became simultaneously an investigative tool, a physiological intervention, and a treatment capable of modifying both phenotype and arrhythmic risk.

Simultaneous high-resolution endocardial–epicardial mapping by Tung and colleagues provided a more detailed reconstruction of scar-related VT three-dimensional architecture. Importantly, the patterns differed according to the underlying disease. Three-dimensional circuits involving non-uniform propagation between myocardial layers predominated in ischaemic cardiomyopathy, whereas two-dimensional circuits confined predominantly to a single surface were more frequent in non-ischemic cardiomyopathy and, when present, were predominantly epicardial.^11^ Thus, epicardial mapping did more than provide another potential ablation target: when integrated with endocardial mapping, it allowed a more complete reconstruction of the three-dimensional VT circuit.

Epicardial mapping also provided a less obvious lesson. After an unsuccessful endocardial procedure, it was initially tempting to assume that the missing component of the circuit must be epicardial. In some patients, however, epicardial mapping also failed to identify electrograms or an activation pattern convincingly explaining the tachycardia. In such cases, renewed and more detailed endocardial mapping could reveal areas of interest that had not been recognized during the initial procedure. In others, the absence of a convincing activation sequence on either surface suggested a predominantly intramyocardial circuit.

Catheter ablation eventually confronted many problems encountered during surgery. Moving VT mapping into the electrophysiology laboratory—away from thoracotomy, cardiopulmonary bypass, and ventriculotomy—made VT induction and mapping easier but did not eliminate its fundamental limitations. Many VTs remained too rapid, unstable, multiple, or non-reproducible for reliable activation and entrainment mapping.

The response was surprisingly familiar: when the tachycardia could no longer guide the procedure, attention shifted towards the substrate. Strategies based on abnormal electrograms and progressively more extensive scar homogenization allowed treatment of otherwise unmappable VTs. In both the surgical and catheter eras, uncertainty about the precise circuit ultimately encouraged broader modification of potentially arrhythmogenic tissue.^12^

More recently, limited scar dechanneling and functional substrate mapping have advanced from this repeated pattern. Rather than requiring sustained VT or assuming that all abnormal myocardium should be eliminated, these strategies dynamically interrogate the substrate during sinus rhythm or ventricular stimulation. Premature stimulation may reveal decremental or concealed slow conduction, whereas propagation-based mapping can identify localized slowing. The question is shifting from where the scar is to which portions display the electrophysiological properties required for re-entry. These approaches may enable more selective ablation without sustained tachycardia.^13–15^

Important challenges remain. Epicardial access carries risk, and complications related to pericardial entry and adjacent structures underscore the importance of patient selection and technical refinement. Moreover, access to both ventricular surfaces does not necessarily reveal the entire re-entrant circuit. Portions of activation may remain hidden within the myocardial wall, and intramural substrate remains incompletely accessible, although advanced imaging and emerging energy-delivery strategies are expanding our ability to identify and reach deeper targets.^8^

Thirty years later, the significance of percutaneous epicardial mapping and ablation extends well beyond providing access to another cardiac surface. Surgery had already demonstrated the complexity of ventricular re-entry. Catheter ablation made its treatment less invasive but initially confined direct mapping largely to the endocardium. Bringing direct epicardial access into the electrophysiology laboratory not only provided a new therapeutic target but also allowed endocardial and epicardial information to be integrated, revealing how differently arrhythmogenic substrates and re-entrant circuits are organized across diseases and how much of their course may remain concealed within the ventricular wall.

What began as an attempt to understand the failure of endocardial ablation ultimately provided a second perspective to understand the three-dimensional ventricular substrate.

## Data Availability

No new data were genarated or analysed in support of this article.
